# Associations Between Advanced Lung Cancer Inflammation Index and Chronic Pain: Insights From National Health and Nutrition Examination Survey (NHANES) 1999–2004

**DOI:** 10.1002/iid3.70053

**Published:** 2024-11-07

**Authors:** Qiqi Huang, Liling Lin, Jingwen Li, Jianwei Lin, Zhaopei Zeng, Yuan Fu, Junxiong Qiu, Junmeng Zheng

**Affiliations:** ^1^ Department of Cardiovascular Surgery, Sun Yat‐Sen Memorial Hospital Sun Yat‐Sen University Guangzhou China; ^2^ Department of Anesthesiology, Sun Yat‐Sen Memorial Hospital Sun Yat‐Sen University Guangzhou China; ^3^ Big Data Laboratory Joint Shantou International Eye Center of Shantou University and the Chinese University of Hong Kong Shantou China

## Abstract

**Introduction:**

Nutrition and inflammation are known factors in chronic pain, but their combined influence is not fully understood. This study investigates the associations between advanced lung cancer inflammation index (ALI) and various types of pain, including joint pain, neck pain, low back pain, and severe headaches and migraines.

**Methods:**

In this cross‐sectional study, a total of 3842 participants were recruited from the National Health and Nutrition Examination Survey (NHANES) from 1999 to 2004. Participants were categorized into three groups based on ALI tertiles: low (≤ 46.982), moderate (> 46.982 and ≤ 70.359), and high (> 70.359). Logistic regression, restricted cubic splines (RCS), and stratified analyses were employed to assess the relationship between ALI and various types of pain.

**Results:**

High ALI consistently correlated with an increased risk of joint pain (fully adjusted odds ratio [OR]: 1.284; 95% confidence interval [CI]: 1.044–1.578) compared to low ALI. However, limited evidence was found in other types of pain. Stratified analyses revealed that high ALI was particularly associated with joint pain in specific demographics, including females (OR: 1.607; 95% CI: 1.205–2.144; *p* = 0.002), individuals aged ≥ 65 years (OR: 1.914; 95% CI: 1.254–2.923; *p* = 0.004), and those with a high school diploma (OR: 1.630; 95% CI: 1.171–2.268; *p* = 0.006). ALI also showed a positive association with multisite pain (*p* < 0.05), with RCS analysis revealing a linear relationship between ALI and joint pain, escalating beyond 57.85.

**Conclusions:**

This study highlights the association between ALI and joint pain, particularly among females and older individuals. Furthermore, ALI may influence the presence of pain at multiple sites.

## Graphical abstract

This study investigates the associations between advanced lung cancer inflammation index (ALI) and various types of pain, including joint pain, neck pain, low back pain, and severe headaches and migraines. Results showed that there was a linear relationship between ALI and joint pain. And ALI may affect the presence of pain at multiple sites.

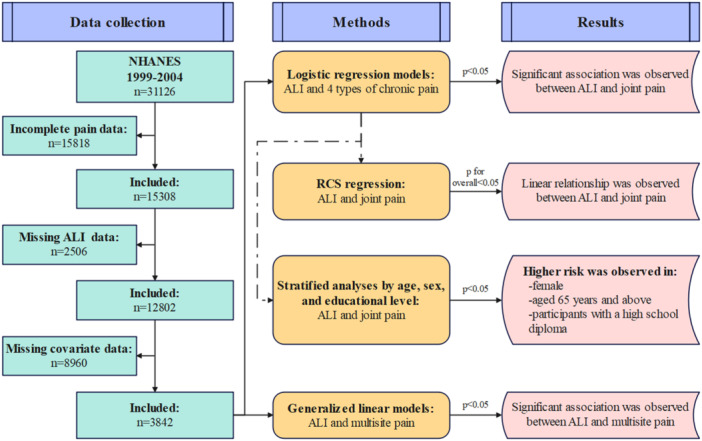

## 1 Introduction

Chronic pain, affecting more than one‐fifth of individuals, is now recognized as a disease in the 11th revision of the International Classification of Diseases (ICD‐11) [1, 2]. Notably, The Global Burden of Disease Study 2019 identified chronic pain as a major contributor to disability, with conditions such as low back pain, neck pain, headache, and other musculoskeletal disorders ranking among the top 20 leading causes of years lived with disability, underscoring the pervasive impact of chronic pain conditions [3].

Understanding the mechanisms underlying chronic pain, identifying at‐risk individuals, and optimizing pain management strategies are paramount in addressing this global health challenge. To this end, the exploration of reliable biomarkers holds immense promise. Previous research has highlighted interleukin‐6 (IL‐6) and the neutrophil‐lymphocyte ratio (NLR) as key players in chronic pain pathophysiology [4, 5, 6, 7]. IL‐6, a pro‐inflammatory cytokine released in response to various stimuli including pain, offers valuable insights into the inflammatory processes underlying chronic pain conditions. Similarly, NLR, as a marker of systemic inflammation, has demonstrated utility in predicting postoperative pain outcomes. In addition to inflammatory markers, nutritional status is increasingly recognized as a critical determinant of pain experience and management. Instruments such as the Malnutrition Universal Screening Tool and the Nutritional Risk Index have been instrumental in identifying individuals at nutritional risk and guiding tailored interventions [8, 9, 10]. However, these conventional biomarkers and nutritional assessment tools may not comprehensively reflect an individual's nutritional and inflammatory status, as inflammation and nutrition often interact and influence each other in complex ways [8, 11, 12].

The interplay between inflammation and nutrition in the context of chronic pain is complex, warranting the exploration of integrated biomarkers that capture both domains comprehensively. One such biomarker of interest is the advanced lung cancer inflammation index (ALI), which incorporates components reflecting both inflammatory (NLR) and nutritional (albumin and BMI) status [13, 14]. ALI has been extensively studied in the context of a number of diseases, including hypertension, heart failure, and Crohn's disease, showing promise as a holistic indicator of health status [13, 15, 16, 17]. However, despite its potential relevance, evidence regarding the association between ALI and chronic pain remains scarce.

To fill this knowledge gap, this study attempts to explore the relationships between ALI and various types of chronic pain, with a particular focus on elucidating age and sex‐specific patterns. Additionally, the study seeks to identify a critical threshold wherein heightened ALI levels correspond to a pronounced escalation in chronic pain risk, thereby providing clinical intervention cues.

## 2 Methods

### 2.1 Study Design and Population

This cross‐sectional study utilized data from the National Health and Nutrition Examination Survey (NHANES), conducted biennially by the National Center for Health Statistics (NCHS). NHANES is a nationally representative survey aimed at assessing the health and nutritional status of individuals in the United States and uses a complex, four‐stage probability sample design to select its participants [18]. All protocols for NHANES were approved by the NCHS Ethics Review Board [19].

**Figure 1:**
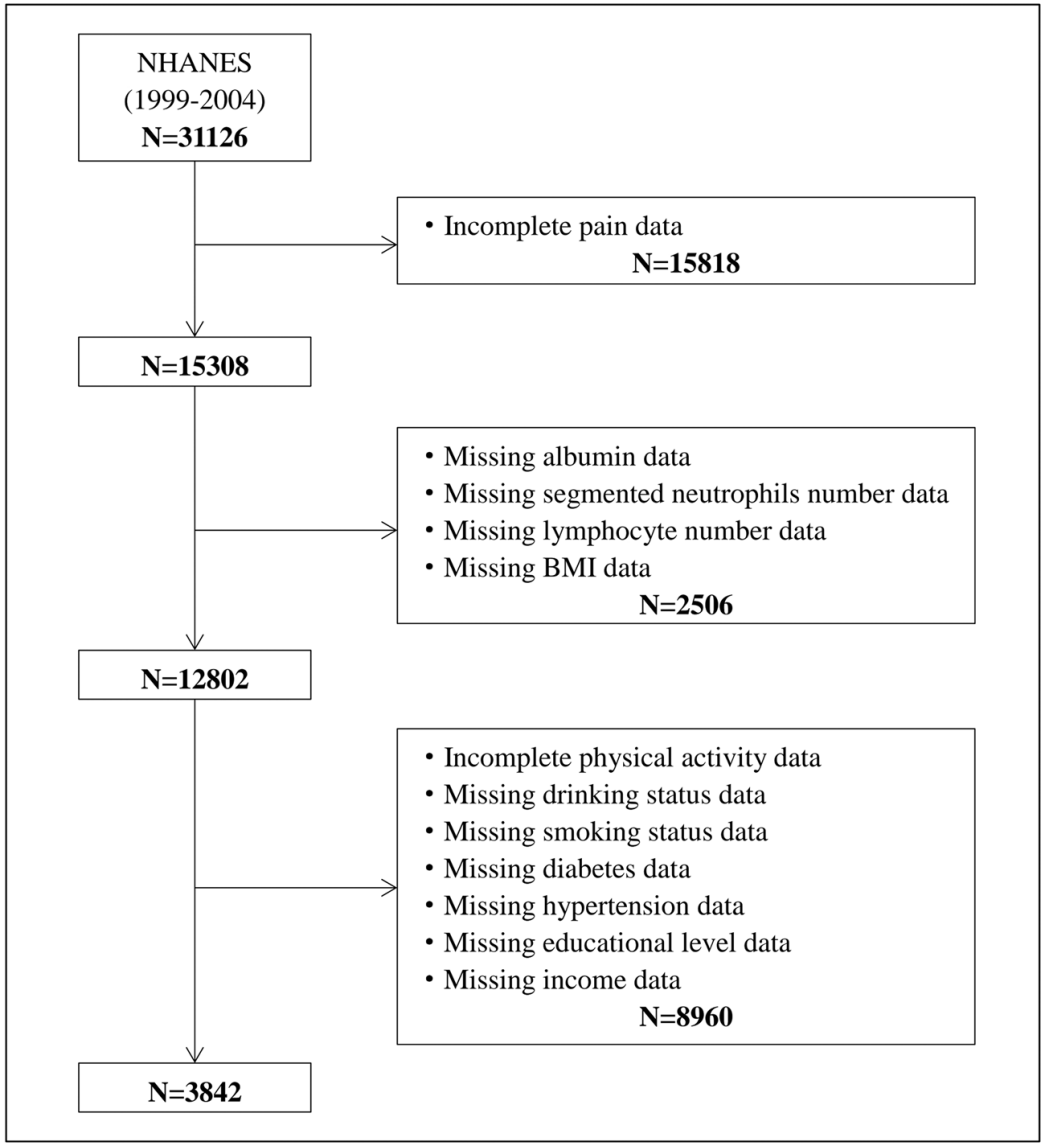
Selection process of eligible participants.

Three consecutive NHANES cycles were included in this study: 1999–2000, 2001–2002, and 2003–2004. Out of 31,126 participants, 15,818 were excluded due to incomplete pain data, 2506 due to missing data on albumin, segmented neutrophils number, lymphocyte number, and BMI, and 8960 due to incomplete covariates. Thus, in the final analysis, there were 3842 participants included in total (Figure 1).

### 2.2 Assessment of ALI

The ALI was calculated using the formula: ALI = BMI × Alb/NLR, where BMI represents body mass index (kg/m²), Alb represents serum albumin (g/dL), and NLR represents the NLR [14, 20]. ALI was categorized into three groups based on tertiles: low (≤ 46.982), moderate (> 46.982 and ≤ 70.359), and high (> 70.359).

### 2.3 Assessment of Pain

Pain assessment was based on the NHANES miscellaneous pain questionnaire. Participants were asked if they had joint pain, neck pain, low back pain, and severe headaches or migraines. For neck pain/low back pain/severe headaches or migraines, the specific questions were “During the past 3 months, did {you/SP} have neck pain/low back pain/severe headaches or migraines?” For joint pain, the specific question was “During the past 12 months, {have you/has SP} had pain, aching, stiffness or swelling in or around a joint? [Do not include neck pain.]”. Participants who answered “yes” were considered to have that type of pain. To account for pain occurring at multiple sites, a new variable called multisite pain (MSP) was created, representing the total number of pain sites experienced by each participant.

### 2.4 Assessment of Covariates

Demographic covariates, lifestyle factors, and personal medical history were extracted from the NHANES questionnaire data. Demographic variables included age, sex, race/ethnicity, educational level, and family poverty income ratio, which serves as a proxy for household income. Race/ethnicity was categorized into distinct groups: Mexican American, Other Hispanic, Non‐Hispanic White, Non‐Hispanic Black, and Other Race. Educational level was classified as more than high school, high school diploma, and less than high school. Lifestyle factors encompassed alcohol use, smoking status, and physical activity. Alcohol use was assessed by self‐reported average drinks per day in the past 12 months. Smoking status was assorted as every day, some days, or not at all. Physical activity was measured across three domains: walked or bicycled (respondents indicated Yes or No), tasks in or around home or yard (respondents indicated Yes or No), and muscle‐strengthening activities over the past 30 days (respondents indicated Yes or No). As for the medical history, diabetes and hypertension were identified by whether participants were informed of having diabetes or hypertension by doctors or health professionals. The use of analgesic pain relievers was assessed by participants' self‐report on whether they regularly took certain prescription or over‐the‐counter pain relievers.

### 2.5 Statistical Analyses

Survey weights recommended by NHANES were applied to account for the complex sampling design and ensure the representativeness of the data [21]. Continuous variables were presented as means with standard deviations, while categorical variables were expressed as numbers and percentages. Logistic regression models were employed to estimate adjusted odds ratios (ORs) and corresponding 95% confidence intervals (CIs) to assess the association between ALI levels (categorized as low, moderate, and high) and the presence of various types of pain (joint pain, neck pain, low back pain, severe headaches or migraines). Generalized linear models were used to analyze the connection between ALI and multisite pain (MSP). Four hierarchical models were constructed to control for potential confounders: Model 1 adjusted for age and sex, Model 2 further included race, educational level, and household income, Model 3 additionally adjusted for smoking status, alcohol use, and medical history, and Model 4 extended adjustments to include physical activity. Additionally, the use of pain relievers was included to perform a sensitivity analysis.

To investigate potential nonlinear associations between ALI and pain and discern any inflection points representing thresholds where changes in ALI levels may notably affect pain outcomes, a restricted cubic spline (RCS) function was utilized. This approach allows for the exploration of complex relationships between variables, capturing nuances that linear models might overlook [22]. To further elucidate whether the impact of ALI on pain is consistent across different demographics, stratified analyses by age, sex, and educational level were conducted. These analyses were performed based on the fully adjusted model (Model 4). All statistical analyses were conducted using R software (version 4.3.1), and a significance level of *p* < 0.05 was considered statistically significant.

## 3 Results

### 3.1 Baseline Characteristics

A total of 3842 participants were included in the investigation, with a mean (SD) age of 48.27 (17.48) years. Among them, 1530 (39.8%) were female and 2312 (60.2%) were male. Participants were categorized into three groups based on ALI tertiles: low (*n* = 1281), moderate (*n* = 1280), and high (*n* = 1281). The racial distribution consisted of 2223 (57.9%) Non‐Hispanic White, 760 (19.8%) Mexican American, 600 (15.6%) Non‐Hispanic Black, 147 (3.8%) other Hispanic, and 112 (2.9%) other race individuals (Table 1).

**Table 1:** Demographic characteristics according to ALI.

	Total	ALI^a^		
		Low (*n* = 1281)	Moderate (*n* = 1280)	High (*n* = 1281)
Age, mean (SD)	48.27 (17.48)	49.77 (19.39)	47.77 (17.06)	47.27 (15.69)
Sex, No. (%)				
Female	1530 (39.8)	555 (43.3)	514 (40.2)	461 (36.0)
Male	2312 (60.2)	726 (56.7)	766 (59.8)	820 (64.0)
Race, No. (%)				
Mexican American	760 (19.8)	198 (15.5)	284 (22.2)	278 (21.7)
Other Hispanic	147 (3.8)	49 (3.8)	49 (3.8)	49 (3.8)
Non‐Hispanic White	2223 (57.9)	878 (68.5)	751 (58.7)	594 (46.4)
Non‐Hispanic Black	600 (15.6)	119 (9.3)	171 (13.4)	310 (24.2)
Other Race	112 (2.9)	37 (2.9)	25 (2.0)	50 (3.9)
Education level, No. (%)				
Less than high school	1105 (28.8)	369 (28.8)	347 (27.1)	389 (30.4)
High school diploma	999 (26.0)	332 (25.9)	326 (25.5)	341 (26.6)
More than high school	1738 (45.2)	580 (45.3)	607 (47.4)	551 (43.0)
Household income, mean (SD)	2.74 (1.62)	2.71 (1.60)	2.77 (1.63)	2.76 (1.63)
BMI(kg/m^2^), mean (SD)	27.92 (5.89)	25.69 (4.74)	27.76 (5.33)	30.30 (6.52)
Smoking status, No. (%)				
Every day	1516 (39.5)	526 (41.1)	512 (40.0)	478 (37.3)
Some days	383 (10.0)	93 (7.3)	148 (11.6)	142 (11.1)
Not at all	1943 (50.6)	662 (51.7)	620 (48.4)	661 (51.6)
Drinks/day^b^, mean (SD)	3.23 (3.04)	3.07 (2.84)	3.20 (2.94)	3.42 (3.32)
Diabetes, No. (%)				
Yes	263 (6.8)	94 (7.3)	82 (6.4)	87 (6.8)
No	3525 (91.7)	1173 (91.6)	1183 (92.4)	1169 (91.3)
Borderline	54 (1.4)	14 (1.1)	15 (1.2)	25 (2.0)
Hypertension, No. (%)				
Yes	1111 (28.9)	363 (28.3)	349 (27.3)	399 (31.1)
No	2731 (71.1)	918 (71.7)	931 (72.7)	882 (68.9)
Walked or bicycled, No. (%)				
Yes	916 (23.8)	301 (23.5)	333 (26.0)	282 (22.0)
No	2863 (74.5)	952 (74.3)	935 (73.0)	976 (76.2)
Unable to do activity	63 (1.6)	28 (2.2)	12 (0.9)	23 (1.8)
Tasks around home/yard, No. (%)				
Yes	2407 (62.6)	790 (61.7)	805 (62.9)	812 (63.4)
No	1381 (35.9)	467 (36.5)	461 (36.0)	453 (35.4)
Unable to do activity	54 (1.4)	24 (1.9)	14 (1.1)	16 (1.2)
Muscle‐strengthening activities, No. (%)				
Yes	942 (24.5)	276 (21.5)	332 (25.9)	334 (26.1)
No	2824 (73.5)	972 (75.9)	929 (72.6)	923 (72.1)
Unable to do activity	76 (2.0)	33 (2.6)	19 (1.5)	24 (1.9)

Abbreviations: ALI, advanced lung cancer inflammation index; BMI, body mass index; SD, standard deviation.

^a^ The ALI was divided into low (≤ 46.982), moderate (> 46.982 and ≤ 70.359), and high (> 70.359) according to the tertiles.

^b^ Average drinks per day in the past 12 months.

### 3.2 Associations Between ALI and Different Types of Pain

**Figure 2:**
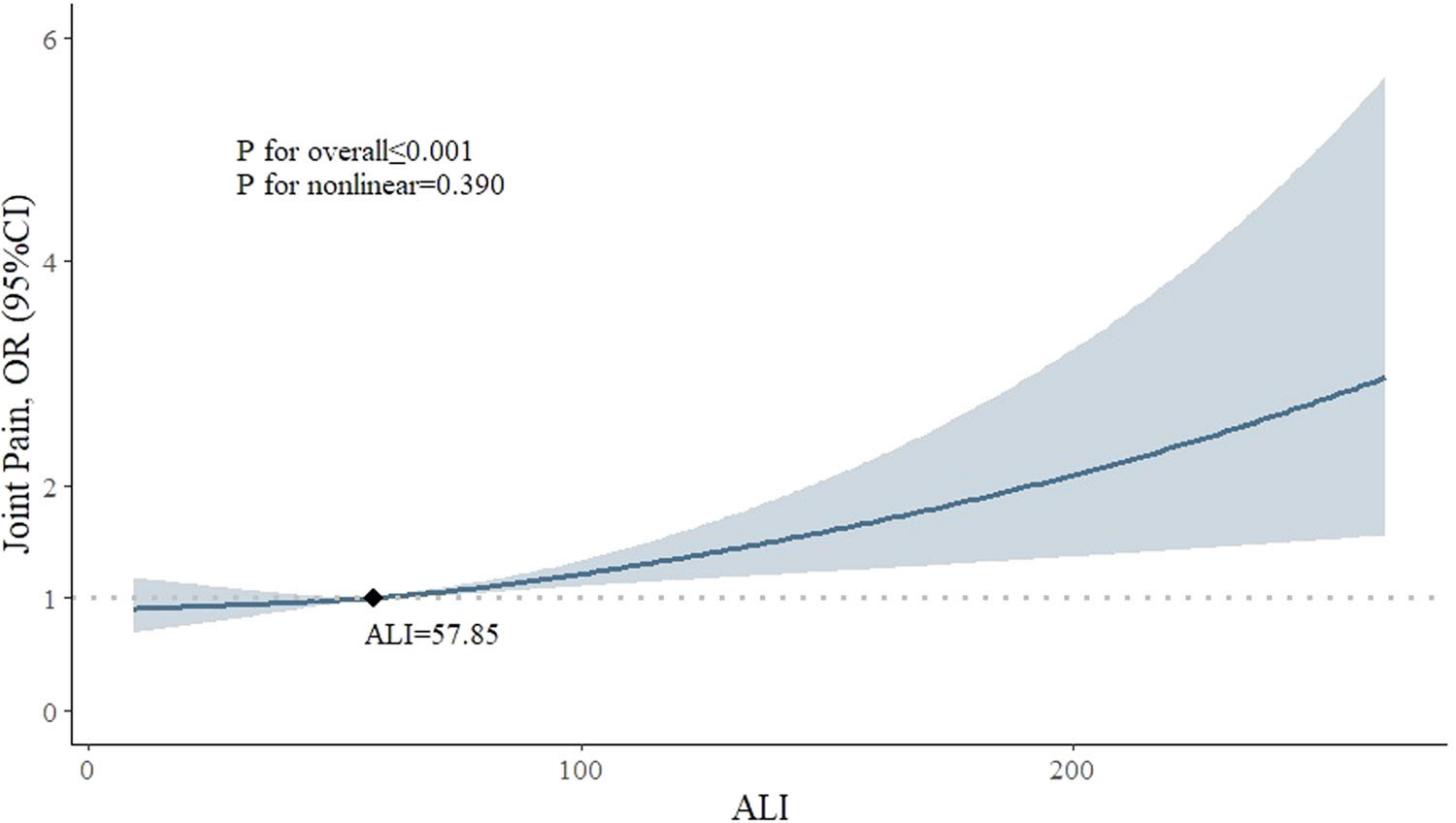
Associations between ALI and joint pain in RCS regression. The model adjusted for age, sex, race, education level, household income, smoking status, alcohol use, hypertension, diabetes, physical activity (walk or bicycle, home task, muscle‐strengthening activity). The sample size for participants aged between 20 and 65 years is 3089, while the sample size for those over 65 years is 753. Regarding sex distribution, the sample size for females and males is 1530 and 2312, respectively. Abbreviations: ALI, advanced lung cancer inflammation index; CI, confidence interval; OR, odds ratio; RCS, restricted cubic spline.

Among all participants (*n* = 3842), 1804 (47.0%) reported joint pain, 747 (19.4%) had severe headaches or migraines, and 824 (21.4%) and 1586 (41.2%) experienced neck pain and low back pain, respectively. Limited evidence was found between ALI and the occurrence of neck pain, low back pain, or severe headaches/migraines in all four models, as illustrated in Table 2. However, ALI showed a significant association with joint pain across all four models. After full adjustment for covariates, participants in the high ALI group had a 28.4% higher risk of experiencing joint pain compared to those in the low ALI group (OR, 1.284; 95% CI, 1.044–1.578). The fully adjusted RCS model suggested a linear relationship between ALI and joint pain (*p* for overall < 0.001, *p* for nonlinear = 0.390). While ALI surpasses 57.85, there is a consistent increase in the risk of joint pain (OR > 1), suggesting this is a critical threshold where heightened ALI levels may warrant clinical attention and intervention (Figure 2). Furthermore, there was a positive significant association between ALI and MSP in all four models (*p* < 0.05), indicating that higher ALI levels may correlate with a greater number of pain sites (Table 3).

**Table 2:** Associations between ALI and Pain.

		Pain, No./Total (%)	OR (95%CI)			
			Model1^a^	Model2^b^	Model3^c^	Model4^d^
Joint pain						
ALI	Low	579/1281 (45.2)	1 [Reference]	1 [Reference]	1 [Reference]	1 [Reference]
	Moderate	588/1280 (45.9)	1.054 (0.911–1.219)	1.084 (0.931–1.261)	1.088 (0.935–1.265)	1.091 (0.935–1.273)
	High	637/1281 (49.7)	1.226 (1.011‐–6)^e^	1.284 (1.053–1.565)^e^	1.280 (1.044–1.568)^e^	1.284 (1.044–1.578)^e^
Neck pain						
ALI	Low	260/1281 (20.3)	1 [Reference]	1 [Reference]	1 [Reference]	1 [Reference]
	Moderate	281/1280 (22.0)	1.009 (0.787–1.292)	1.044 (0.890–1.346)	1.050 (0.813–1.356)	1.055 (0.811–1.372)
	High	283/1281 (22.1)	0.983 (0.733–1.318)	1.008 (0.747–1.360)	1.021 (0.753–1.383)	1.026 (0.751–1.402)
Low back pain						
ALI	Low	535/1281 (41.8)	1 [Reference]	1 [Reference]	1 [Reference]	1 [Reference]
	Moderate	505/1280 (39.5)	0.928 (0.763–1.129)	0.976 (0.799–1.192)	0.982 (0.803–1.201)	0.985 (0.800–1.214)
	High	546/1281 (42.6)	1.048 (0.829–1.324)	1.108 (0.868–1.414)	1.104 (0.860‐–7)	1.105 (0.851–1.435)
Severe headaches or migraines						
ALI	Low	244/1281 (19.0)	1 [Reference]	1 [Reference]	1 [Reference]	1 [Reference]
	Moderate	239/1280 (18.7)	1.002 (0.815–1.232)	1.049 (0.852–1.291)	1.057 (0.850–1.315)	1.066 (0.858–1.324)
	High	264/1281 (20.6)	1.143 (0.899–1.470)	1.179 (0.903–1.541)	1.192 (0.904–1.572)	1.204 (0.908‐–5)

Abbreviations: ALI, advanced lung cancer inflammation index; CI, confidence interval; OR, odds ratio.

^a^ Adjusted for age, sex.

^b^ Adjusted for age, sex, race, education level, household income.

^c^ Adjusted for age, sex, race, education level, household income, smoking status, alcohol use, hypertension, diabetes.

^d^ Adjusted for age, sex, race, education level, household income, smoking status, alcohol use, hypertension, diabetes, physical activity (walk or bicycle, home task, muscle‐strengthening activity).

^e^ *p* < 0.05.

**Table 3:** Associations between ALI and MSP.

	β (95%CI), *p*‐value			
	Model1^a^	Model2^b^	Model3^c^	Model4^d^
MSP	0.001 (0.000‐0.003), 0.02^e^	0.002 (0.001‐0.003), 0.005^e^	0.002 (0.001‐0.003), 0.007^e^	0.002 (0.001‐0.003), 0.008^e^

Abbreviations: ALI, advanced lung cancer inflammation index; CI, confidence interval; MSP, multisite pain.

^a^ Adjusted for age, sex.

^b^ Adjusted for age, sex, race, education level, household income.

^c^ Adjusted for age, sex, race, education level, household income, smoking status, alcohol use, hypertension, diabetes.

^d^ Adjusted for age, sex, race, education level, household income, smoking status, alcohol use, hypertension, diabetes, physical activity (walk or bicycle, home task, muscle‐strengthening activity).

^e^ *p* < 0.05.

### 3.3 Stratified Analyses

**Figure 3:**
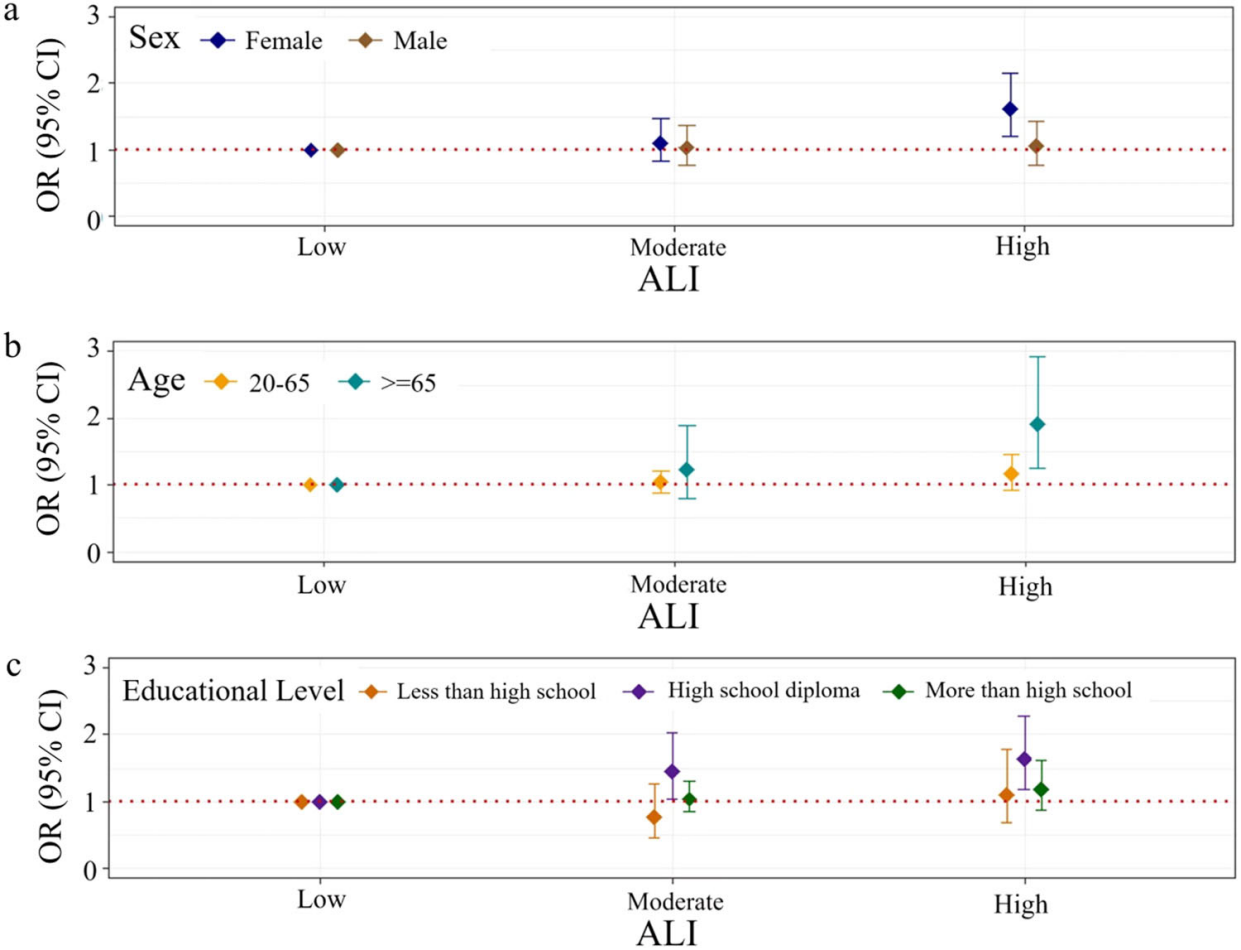
Subgroup analyses for the association between ALI and joint pain. a. Subgroup analyses by sex; b. Subgroup analyses by age; c. Subgroup analyses by educational level. The sample size for female participants is 1530, while the sample size for male participants is 2312. Within the age groups, the sample size for those aged between 20 and 65 years and those over 65 years is 3089 and 753, respectively. Regarding the distribution of educational level, 1105 participants are less than high school, 999 have high school diplomas, and 1738 are more than high school. Abbreviations: ALI, advanced lung cancer inflammation index; CI, confidence interval; OR, odds ratio.

The results of the stratified analyses revealed a significant association between ALI and joint pain primarily among females, older individuals, and those with a high school diploma. Specifically, females (OR: 1.607; 95% CI: 1.205–2.144; *p* = 0.002), individuals aged 65 years and above (OR: 1.914; 95% CI: 1.254–2.923; *p* = 0.004), and participants with a high school diploma (OR: 1.630; 95% CI: 1.171–2.268; *p* = 0.006) in the high ALI group exhibited a heightened likelihood of experiencing joint pain compared to their counterparts in the low ALI group (Figure 3). These findings suggest that females, older individuals, and those with a high school diploma are particularly susceptible to developing joint pain when exposed to high ALI values.

### 3.4 Sensitivity Analyses

Similar patterns were observed upon including pain relievers as a confounder in the sensitivity analysis. Participants in the high ALI group exhibited a 29.7% increased risk of experiencing joint pain compared to those in the low ALI group (OR, 1.297; 95% CI, 1.055–1.595). Furthermore, a significant positive relationship was found between ALI and MSP (*p* < 0.05) (Supporting Information S1: eTable 1 and eTable 2).

## 4 Discussion

In this research, we examined the relationship between the ALI and different types of pain. Our findings suggest that individuals with high ALI may have an elevated risk of experiencing joint pain compared to those with low ALI levels. Moreover, stratified analyses revealed a positive association between ALI and joint pain specifically among female and older participants. Female and older individuals in the highest quartile of ALI were 60.7% and 91.4% more likely to report joint pain, respectively, compared to those in the lowest quartile. However, significant associations between ALI and neck pain, low back pain, or severe headaches/migraines were not observed.

Previous studies have established links between inflammatory and nutritional status and joint pain. For instance, Cai et al. [23] found a close association between NLR and the development of osteoarthritis in knee osteoarthritis patients, highlighting the role of inflammation in joint dysfunction. Similarly, Elsayed et al. [24] reported higher NLR levels in patients with rheumatoid arthritis, with a significant proportion experiencing arthralgia and morning stiffness. Furthermore, research by Heo et al. [25] and Raud et al. [26] demonstrated associations between BMI and joint pain, suggesting that obesity may exacerbate its prevalence, particularly in knee osteoarthritis. As far as we know, this is the first study to explore the relationship between ALI and joint pain, where ALI encompasses both inflammatory and nutritional components. Our findings, supported by the reference value derived from the RCS model, suggest a positive association between ALI and joint pain, particularly when ALI exceeds 57.85. This threshold could serve as a valuable reference for healthcare professionals when assessing joint pain.

Several potential biological mechanisms may contribute to the connection between elevated ALI and heightened risk of joint pain. Inflammatory mediators are known to play a pivotal part in arthritis pathogenesis, characterized by chronic inflammation marked by increased pro‐inflammatory cytokine levels, including interleukin‐1β (IL‐1β) and tumor necrosis factor‐α [27]. These cytokines foster synovial inflammation, precipitating the release of matrix metalloproteinases (MMPs) and other proteolytic enzymes, thereby contributing to cartilage degradation and joint destruction [28]. Additionally, chronic synovial inflammation can stimulate osteoclast activity, resulting in bone resorption and joint erosion, thereby exacerbating joint pain and dysfunction. Recent research has also revealed that pain is regulated by artemin (ARTN) and TAFA4. ARTN, a member of the glial cell line‐derived neurotrophic factor (GDNF) family regulates the sensitivity of thermal nociceptors and hyperalgesia caused by inflammation. By binding to GFRα3, one of the GDNF family receptors, ARTN could exert an impact on intracellular signaling pathways that are crucial in the pathophysiology of bone pain and migraine [29, 30, 31]. As for TAFA4, a member of the TAFA chemokine‐like ligand or FAM19A family, was implicated as a protective role for chemically induced neuro‐inflammatory pain and spinal cord pain [32]. Besides inflammation, oxidative stress and metabolic abnormalities have also been implicated in arthritis pathogenesis. Oxidative stress, typified by an imbalance between reactive oxygen species (ROS) production and antioxidant defense mechanisms, can incite inflammation and tissue damage in the joints [29]. Given that ALI serves as a composite marker of inflammation and nutritional status, it mirrors systemic inflammation and oxidative stress levels, potentially amplifying the inflammatory processes underlying joint diseases.

Stratified analyses revealed a significant association between ALI and joint pain among female and older participants. These findings imply a potential role of sex hormones in the underlying mechanism linking ALI to joint pain. Evidence suggests that estrogen fluctuations may influence pain perception, with stable hormone levels potentially serving as protective factors. Declines in estrogen levels, such as those experienced during menopause, may contribute to more severe musculoskeletal pain and chronic arthralgia [30, 31, 32]. Estrogen, predominantly found in females, modulates pain processing pathways and inflammatory responses within the joint microenvironment. Experimental studies also confirm the analgesic properties of estrogen, mediated by its regulation of neurotransmitter receptors and endogenous opioidergic systems [33]. This association underscores the pivotal role of estrogen in joint pain pathogenesis, highlighting avenues for future research and therapeutic exploration. The observation that individuals with a high school diploma are particularly vulnerable to developing joint pain when exposed to high ALI values resonates with existing research indicating the influence of occupational factors on musculoskeletal health. Occupations commonly associated with high school diploma holders often involve manual labor and repetitive tasks, which can impose significant stress on joints, increasing the risk of joint pain and related musculoskeletal disorders [34]. Further prioritization for understanding and addressing the specific occupational challenges of individuals with a high school diploma to effectively prevent and manage joint pain is needed.

While our study revealed significant associations between ALI and joint pain, we did not find similar links with other types of pain. This absence of significant findings may be attributed to limitations in sample size, which could have compromised statistical power [35]. Nonetheless, the observed positive association between ALI and MSP hints at a potential connection between ALI and other types of pain, warranting further investigation.

This study benefited from the comprehensive use of NHANES data, which employs a sophisticated, multistage, probability sampling design in the United States, ensuring a nationally representative sample. As a result, the findings of this study can be reliably extrapolated to the broader population of the country. Notably, this research marks the inaugural investigation into the relationship between ALI and various types of pain, thereby contributing novel insights to the field.

However, several limitations warrant consideration. Firstly, as this study is focused on the United States, the generalizability of its findings to regions with different population distributions, such as Asia and Africa, may be limited. Secondly, due to its cross‐sectional design, this study captured data at a single time point, precluding the assessment of pain duration and changes in ALI over time. Additionally, while the study identified an association between ALI and joint pain, it did not establish causality. Lastly, the reliance on self‐reported pain assessments may introduce potential biases, undermining the validity of the findings.

In summary, our study highlights a significant association between elevated ALI and joint pain, particularly among female and older participants. While providing novel insights into the interplay of inflammation, nutrition, and pain, the study underscores the need for further research to elucidate causality and explore potential therapeutic implications.

## Author Contributions

**Qiqi Huang:** conceptualization, data curation, formal analysis, investigation, writing–original draft, writing–review and editing. **Liling Lin:** conceptualization, data curation, formal analysis, investigation, writing–original draft, writing–review and editing. **Jingwen Li:** conceptualization, data curation, formal analysis, investigation, writing–original draft, writing–review and editing. **Jianwei Lin:** data curation, formal analysis, investigation. **Zhaopei Zeng:** data curation, formal analysis. **Yuan Fu:** supervision. **Junxiong Qiu:** supervision. **Junmeng Zheng:** funding acquisition, supervision.

## Ethics Statement

All protocols for NHANES were approved by the NCHS Ethics Review Board.

## Conflicts of Interest

The authors declare no conflicts of interest.

## Supporting information

Supporting information.

## Data Availability

The original data used in this study were from the publicly available database (https://wwwn.cdc.gov/nchs/nhanes/Default.aspx). All the data used can be obtained from a reasonable request to the corresponding authors.
