# Supplementary material for: Associations Between Advanced Lung Cancer Inflammation Index and Chronic Pain: Insights From National Health and Nutrition Examination Survey (NHANES) 1999–2004
Source: Immun Inflamm Dis. 2024 Nov 7;12(11):e70053. doi: 10.1002/iid3.70053 (PMC11542284; doi:10.1002/iid3.70053)
Supplement: Supplementary file 1 — Supporting information. [file IID3-12-e70053-s001.docx]

**eTable 1.** Associations between ALI and Pain further adjusted for analgesic pain relievers


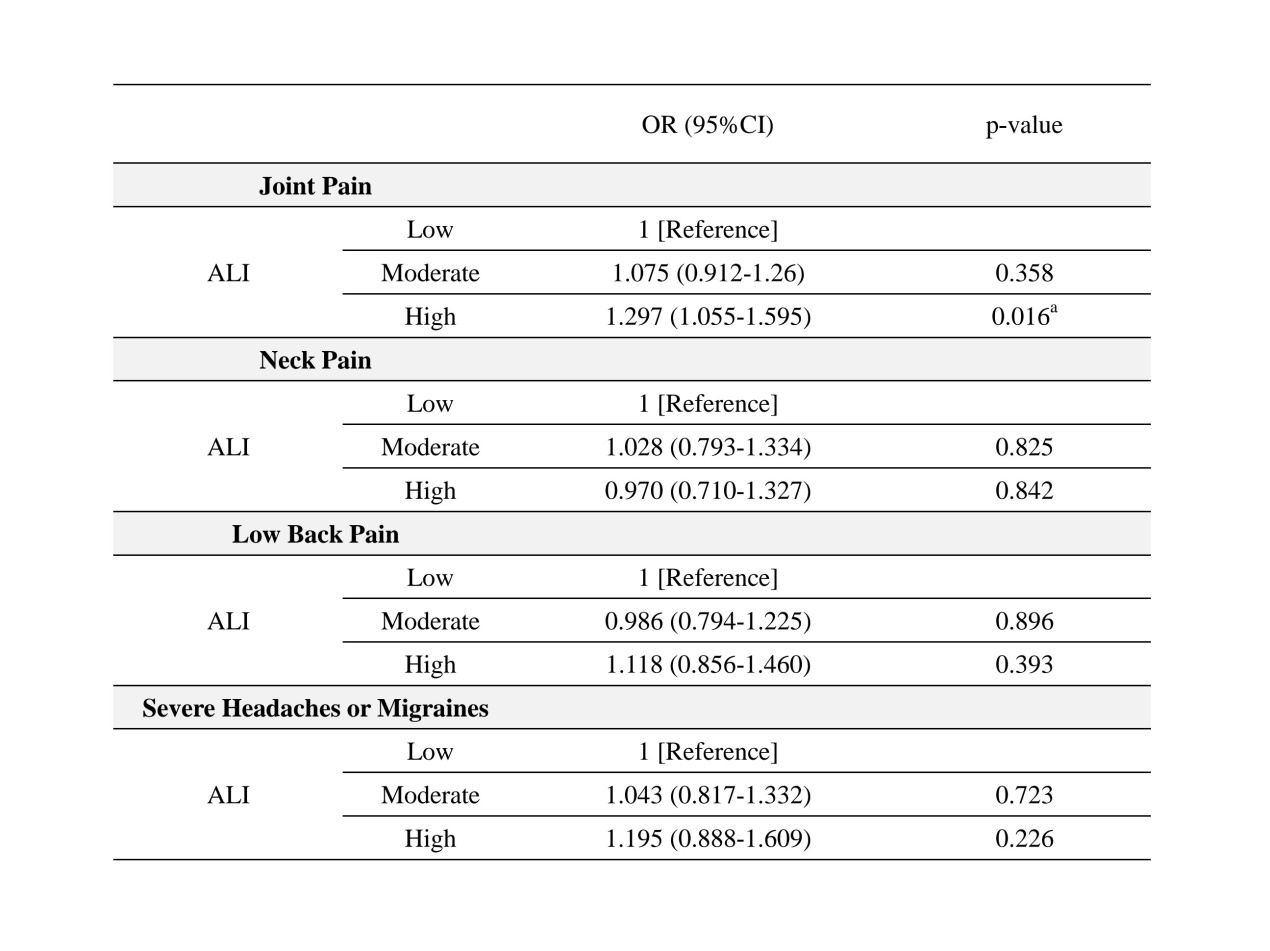


Abbreviations: ALI, advanced lung cancer inflammation index; OR, odds ratio; CI, confidence interval.

Adjusted for age, sex, race, education level, household income, smoking status, alcohol use, hypertension, diabetes, physical activity (walk or bicycle, home task, muscle-strengthening activity), analgesic pain relievers.

^a^p < 0.05

**eTable 2.** Associations between ALI and MSP further adjusted for analgesic pain relievers


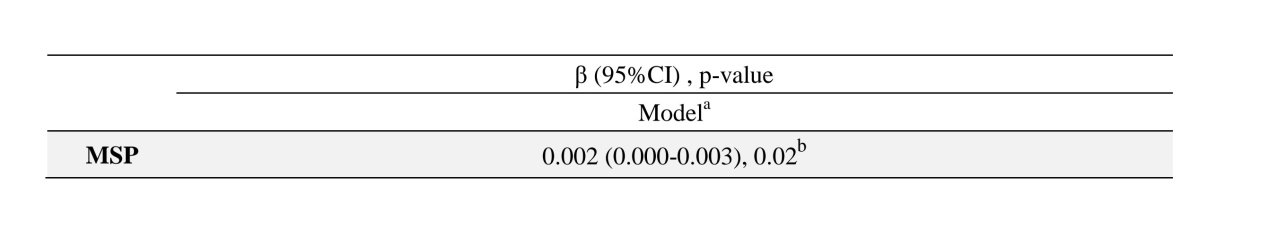


Abbreviations: ALI, advanced lung cancer inflammation index; OR, odds ratio; CI, confidence interval.

Adjusted for age, sex, race, education level, household income, smoking status, alcohol use, hypertension, diabetes, physical activity (walk or bicycle, home task, muscle-strengthening activity), analgesic pain relievers.

^a^p < 0.05
